# Localization of PPAR isotypes in the adult mouse and human brain

**DOI:** 10.1038/srep27618

**Published:** 2016-06-10

**Authors:** Anna Warden, Jay Truitt, Morgan Merriman, Olga Ponomareva, Kelly Jameson, Laura B. Ferguson, R. Dayne Mayfield, R. Adron Harris

**Affiliations:** 1 Waggoner Center for Alcohol and Addiction Research, The University of Texas at Austin, Austin, TX 78712, United States; 2 The Institute for Neuroscience, The University of Texas at Austin, Austin, TX 78712, United States

## Abstract

Peroxisome proliferator-activated receptors (PPARs) are nuclear hormone receptors that act as ligand-activated transcription factors. PPAR agonists have well-documented anti-inflammatory and neuroprotective roles in the central nervous system. Recent evidence suggests that PPAR agonists are attractive therapeutic agents for treating neurodegenerative diseases as well as addiction. However, the distribution of PPAR mRNA and protein in brain regions associated with these conditions (i.e. prefrontal cortex, nucleus accumbens, amygdala, ventral tegmental area) is not well defined. Moreover, the cell type specificity of PPARs in mouse and human brain tissue has yet to be investigated. We utilized quantitative PCR and double immunofluorescence microscopy to determine that both PPAR mRNA and protein are expressed ubiquitously throughout the adult mouse brain. We found that PPARs have unique cell type specificities that are consistent between species. PPARα was the only isotype to colocalize with all cell types in both adult mouse and adult human brain tissue. Overall, we observed a strong neuronal signature, which raises the possibility that PPAR agonists may be targeting neurons rather than glia to produce neuroprotection. Our results fill critical gaps in PPAR distribution and define novel cell type specificity profiles in the adult mouse and human brain.

Peroxisome proliferator-activated receptors (PPARs) are ligand-activated transcription factors belonging to the nuclear hormone receptor superfamily^1^. PPARs regulate gene expression by binding to specific DNA sequence elements within the promoter region of target genes called PPAR response elements (PPREs)^2^. Upon activation by their ligands, PPARs heterodimerize with retinoid X receptors, then bind to PPREs, and act as ligand-regulated transcription factors^3^. There are three known PPAR isotypes (PPARα, PPARβ/δ, and PPARγ) that have been identified in various species and are structurally homologous^4^. Different PPAR isotypes display distinct physiological functions depending on their differential ligand activation and tissue distribution^3^. Moreover, PPARα, PPARβ/δ, and PPARγ show unique tissue distribution in the peripheral nervous system and select regions of the central nervous system in adult rat brain^5^. However, cell-type specificity of PPARs in the adult mouse brain and human brain have not been investigated.

PPARs primarily act as lipid sensors and regulators of lipid metabolism (for review see^6^); however, PPARs also act to inhibit proinflammatory gene expression. Specifically, PPARs have been shown to antagonize the actions of proinflammatory transcription factors nuclear factor-κB (NF-κB) and activator protein 1 (AP-1)^2^. Due to PPARs anti-inflammatory and potentially neuroprotective effects, there is an increased interest in PPAR agonists for the treatment of neurodegenerative diseases such as Alzheimer’s, Parkinson’s, and Huntington’s disease as well as ischemic brain injury, multiple sclerosis, and even addiction^4^,^7^. To date, PPARγ has been the main focus of studies investigating the role of PPAR agonists in neuroinflammation and their therapeautic potential—mainly for treating Alzheimer’s disease^4^.

The expression of PPAR isotypes has been investigated by immunohistochemistry (IHC), quantiative PCR (qPCR), and *in situ* hybridization^8^,^9^,^10^,^11^,^12^,^13^. Yet, there are critical gaps in the literature in brain regions crucial to neurodegenerative diseases and addiction (i.e. prefrontal cortex (PFC), nucleus accumbens (NAC), amygdala (AMY) and ventral tegmental area (VTA)) on both the mRNA and protein level. Cell type specificities of PPARs have also been previously investigated *in situ* and *in vitro*^9^,^14^,^15^,^16^,^17^. However, there have been few studies investigating the cell type specificities of PPAR isotypes in rat brain tissue that were not restricted to a single brain region or did not rely solely on morphology^5^,^9^. Additionally, to the best of our knowledge, there have been no comprehensive studies of PPAR cell type specificity in human brain tissue other than PPARβ/δ and PPARγ in neuroblastoma cell lines^18^,^19^.

The presence of the different PPAR isotypes in specific cell types is still somewhat conflicting, in particular for astrocytes and microglia. The presence of all PPARs in neurons has been well documented *in vitro* and by morphology. PPARβ/δ has been found in neurons in numerous brain areas and in culture^5^,^9^,^14^,^20^. PPARs α and γ have been localized in neuronal culture and to more restricted brain areas^5^. Additionally, PPAR agonist adminstration (α, β/δ, and γ) results in an increase in genes preferentially expressed in neurons^21^. Yet, the definitive presence of PPARs in glia remains elusive. The presence of all PPAR isotypes has been documented in primary astrocyte culture^14^. However, on the protein level several studies have found conflicting evidence as to the presence or absence of PPAR isotypes in astrocytes in brain tissue^5^,^20^—highlighting that the *in vitro* model does not completely mimic the *in vivo* one, lacking the biomolecular interactions among cellular components that are present *in vivo*. Furthermore, despite the well-documented anti-inflammatory effects of PPAR agonists, there is only one study investigating the localization of PPARγ in microglia culture^22^. The presence of PPARα or PPARβ/δ in microglia remains unexplored. PPAR isotype cell type specificity has been shown to be dependent on brain area and developmental age^14^,^20^,^23^. Thus, the success of PPAR agonists to produce neuroprotective changes in a brain region-dependent manner necessitates an enhanced understanding of PPAR isotype cell type specificity in brain tissue.

The aim of this present work was to fill critical gaps in PPAR expression data by providing a more detailed distribution map of PPAR isotype mRNA and protein in specific brain regions that are implicated in neurodegenerative diseases and addiction. Importantly, we sought to resolve conflicting studies concerning the cell type specificity of PPAR isotypes in mouse brain as well as provide novel cell type specificity profiles in human postmortem brain tissue. Utilizing double immunofluorescence, we show that only PPARα colocalizes with all cell types in both adult mouse and adult human brain tissue. PPARβ/δ appears to be mostly present in neurons in grey matter across brain regions. PPARγ is only present in neurons and astrocytes despite the numerous studies that observed a reduction in microglial activation after PPARγ agonist administration. After lipopolysaccharide (LPS) injection, to induce a strong neuroimmune response, we examined PPAR isotype expression as well as colocalization with microglia. After LPS treatment, we observed no change in overall PPAR isotype expression. However, we found that after LPS administration, PPARγ changes its cell-type specificity to weakly colocalize with microglia in a brain region-dependent manner. The strong neuronal signature of all PPAR isotypes was surprising and suggests a new role for PPAR agonists in targeting neurons rather than glial cells. These findings will enable future studies to select cell type specific PPAR agonists to provide targeted neuroprotective treatments for neurodegenerative diseases.

## Results

### PPAR isotype mRNA expression in adult mouse brain

**Figure 1:**
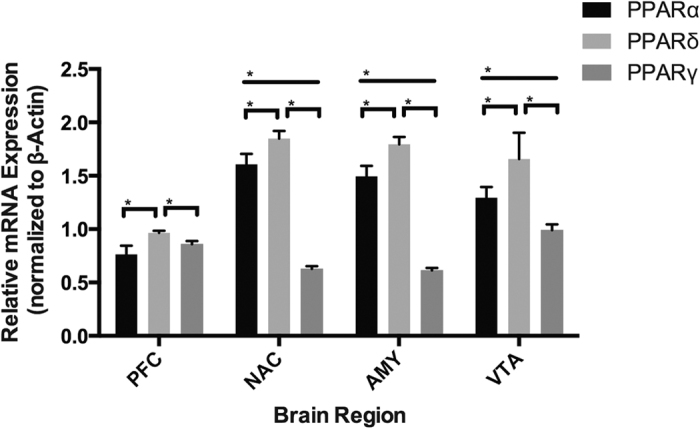
qPCR analysis for PPAR isotype expression in adult mouse brain. β-Actin was used as an endogenous control to normalize target gene mRNA levels.

PPAR isotype mRNA was ubiquitously expressed in all brain regions (Fig. 1). Consistent with the only previous study of PPAR isotype mRNA in coarser brain regions^8^, PPARβ/δ was more highly expressed than PPARα and PPARγ across all brain regions (one-way ANOVA F(2,177) = 1238, p < 0.0001). Additionally, PPARα was more highly expressed than PPARγ in all brain regions except the prefrontal cortex (Tukey HSD, p < 0.001). To complement the mRNA expression profile, we next sought to determine not only the distribution but also the cell type specificity profiles of PPAR isotype protein utilizing immunohistochemical techniques.

### PPAR isotype protein distribution in adult mouse brain

**Figure 2:**
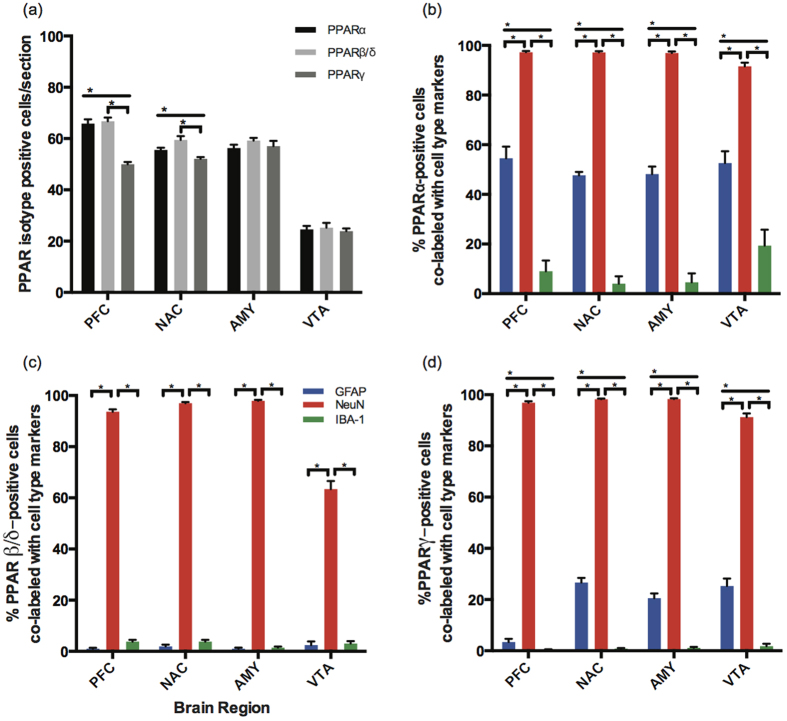
(**a**) Quantification of PPAR isotype distribution in subregions of the adult mouse brain. (**b–d**) Cell type specificity and distribution of PPAR isotype colocalization in adult mouse brain. Data are represented as mean + SEM, n = 5 per group, *p < 0.001.

All PPAR isotypes were weakly- to moderately-detected in all brain regions (Table 1, Fig. 2a), consistent with the ubiquitous distribution seen in the qPCR data above. PPARα and PPARβ/δ were significantly higher expressed than PPARγ but only in the PFC and NAC (two-way ANOVA F(2,492) = 7.405, p = 0.0006) (Fig. 2a). Previous findings indicated PPARβ/δ as the most widely and highly expressed isotype across brain regions on both the mRNA and protein level^5^,^9^, however, our study detected only significant differences in mRNA expression (p < 0.0001) with no signficant difference between PPARα and PPARβ/δ protein expression across all brain regions (p = 0.84). The VTA displayed significantly lower expression compared to other brain regions for all isotypes (F(3,492) = 532.026, p < 0.00001) revealed by two-way ANOVA (Fig. 2a).

### PPAR isotypes demonstrate unique cell type specificity in adult mouse brain

To determine the cell type specificity of PPAR isotypes as well as the relative expression of colocalized cells, we utilized dual-labeled immunofluorescence. We observed a general trend that PPAR isotypes have a strong neuronal signature in all brain regions; whereas, astrocytes and microglia appeared to have more varied expression of PPAR isotype colocalization depending on the isotype and brain region of interest. This suggested that PPAR isotype cell type specificity varies not only between isotypes but also between brain regions.

### PPARα

**Figure 3:**
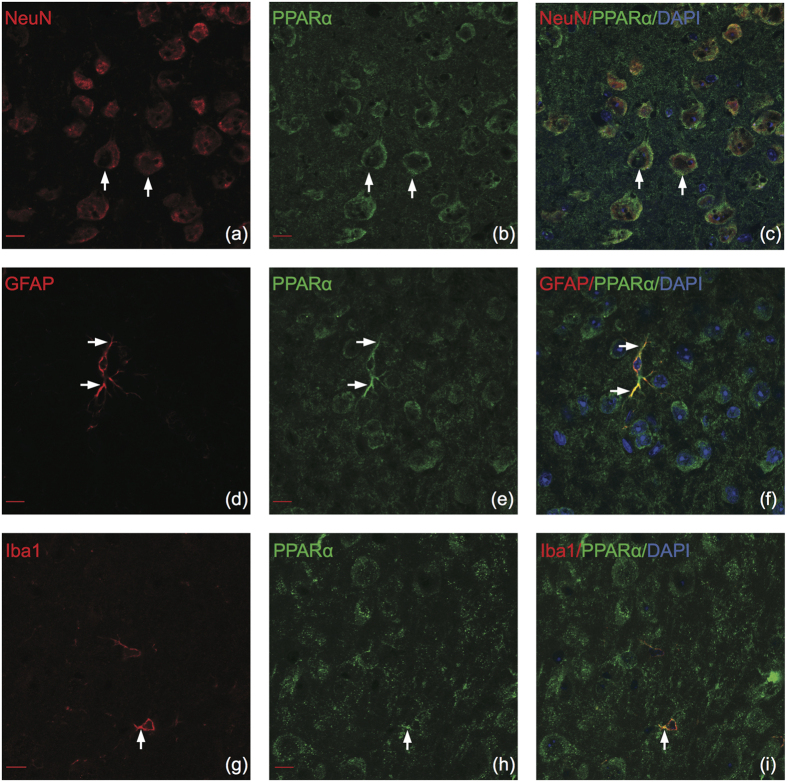
PPARα colocalizes with neurons, astrocytes and microglia in the adult mouse brain. Representative images of cell type specific stains color-coded in red (left panels), PPARα color-coded in green (center panels), and merged images (right panels). (**a–c**) PPARα colocalizes with NeuN in the nucleus. (**d–f**) PPARα colocalizes with GFAP in the cell body and processes. (**g–i**) PPARα colocalizes with Iba1 in processes but not the cell body. Arrows indicate positive colocalization. Confocal (63×), scale bar = 10 μm.

PPARα displayed a strong neuronal signature, over 90% of PPARα positive cells were colabeled with neurons in all brain regions (Fig. 2b). Moreover, PPARα colocalizes significantly more with neurons (NeuN) than with astrocytes (GFAP) or microglia (Iba1) (two-way ANOVA F(2,168) = 1463, p = < 0.0001). Confocal microscopy confirmed that PPARα colocalized with neurons, primarily in the nucleus (representative dual-labeled immunohistochemical images in Fig. 3a-c). We observed moderate coexpression of astrocytes and PPARα, with the highest percentage of colocalization in the PFC (Fig. 2b). PPARα colocalizes with astrocytes in the cell body as well as in astrocytic processes (Representative images in Fig. 3d–f). A significant difference was found between PPARα colocalization with astrocytes versus microglia (two-way ANOVA F(2,168) = 1463, p = < 0.0001), suggesting that PPARα is potentially preferentially expressed in astrocytes. PPARα weakly colocalized with microglia (Fig. 3g–i), with <20% of PPARα positive cells colocalizing with microglia in the VTA and <10% colocalizing in the PFC, NAC, or AMY (Fig. 2b). There was a significant interaction effect between brain region and microglia colocalization (two-way ANOVA F(6,168) = 5.085, p < 0.0001).

### PPARβ/δ

**Figure 4:**
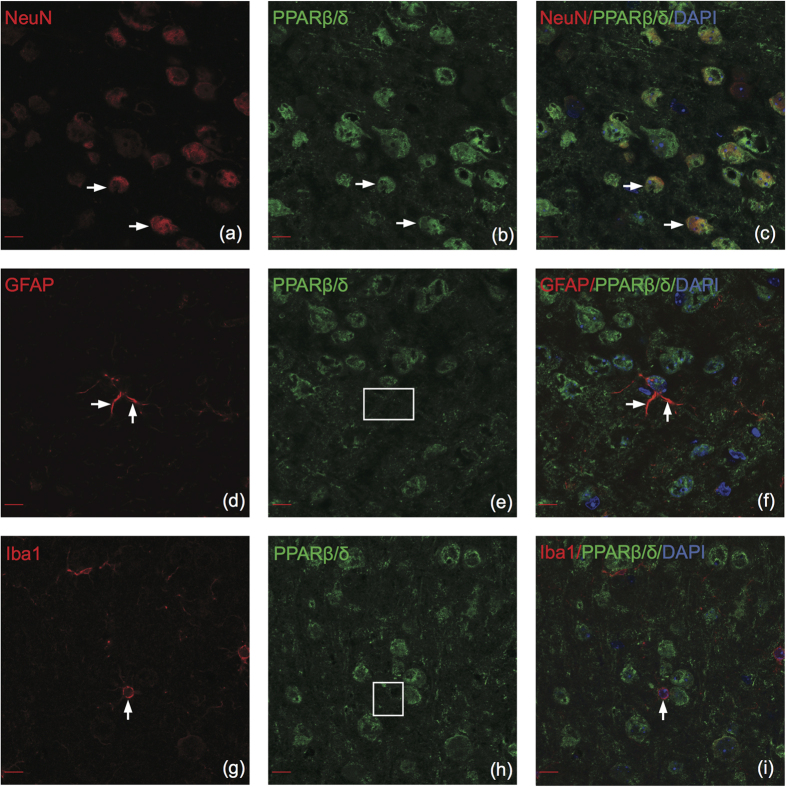
PPARβ/δ colocalizes with neurons in the adult mouse brain. Note the clear absence of colocalization between PPARβ/δ and astrocytes/microglia. Representative images of cell type specific stains color-coded in red (left panels), PPARβ/δ color-coded in green (center panels), and merged images (right panels). (**a–c**) PPARβ/δ colocalizes with NeuN in the nucleus and cytoplasm. (**d–f**) PPAR β/δ does not colocalize with GFAP in grey matter. (**g–i**) PPAR β/δ does not colocalize with Iba1. Arrows indicate positive colocalization examples and boxes represent negative colocalization. Confocal (63×), scale bar = 10 μm.

Similar to PPARα, PPARβ/δ displayed a strong neuronal signature with high percentages of colocalization between PPARβ/δ and NeuN in the PFC, NAC and AMY and moderate colocalization in the VTA (Fig. 2c). PPARβ/δ colocalized significantly more with neurons than with astrocytes or microglia (two-way ANOVA F(2,180) = 7486.99, p = < 0.001). Confocal microscopy confirmed colocalization between PPARβ/δ and neurons, primarily in the nucleus (Fig. 4a–c). In contrast, PPARβ/δ did not appear to colocalize with astrocytes in grey matter of any brain region, confocal microscopy confirmed this negative finding (Fig. 4d–f). Confocal microscopy showed that PPARβ/δ does not colocalize with microglia (Fig. 4g–i). There was no significant difference between PPARβ/δ colocalization with astrocytes and microglia (Tukey HSD, p = 0.69), consistent with our negative colocalization findings.

### PPARγ

**Figure 5:**
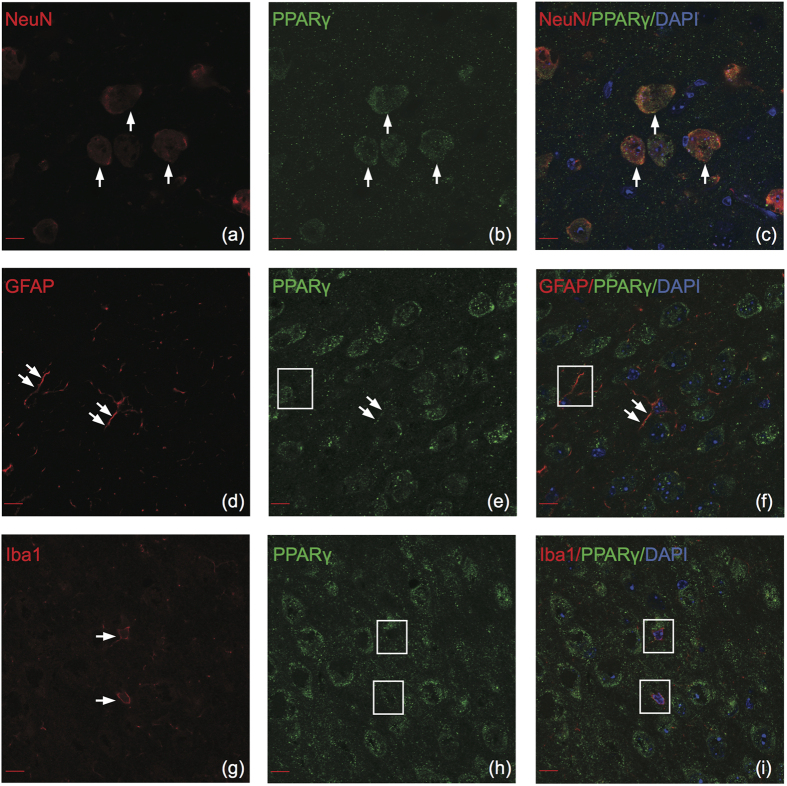
PPARγ colocalizes with neurons and astrocytes in the adult mouse brain. Note the clear absence of colocalization between PPARγ and microglia. Representative images of cell type specific stains color-coded in red (left panels), PPARγ color-coded in green (center panels), and merged images (right panels). (**a–c**) PPARγ colocalizes with NeuN in the nucleus and cytoplasm. (**d–f**) PPARγ colocalizes with GFAP only in the astrocytic processes. (**g–i**) PPARγ does not colocalize with Iba1. Arrows indicate positive colocalization examples and boxes represent negative colocalization. Confocal (63×), scale bar = 10 μm.

Similar to both previous isotypes, PPARγ also had a strong neuronal signature (Fig. 5a–c). PPARγ was more highly expressed in neurons than in astrocytes or microglia (Fig. 2d) (two-way ANOVA F(2,168) = 5361.10, p < 0.0001). PPARγ demonstrated differential colocalization with astrocytes depending on the brain region, with the highest percentage of colocalization in the NAC (26.7% + 0.5) and the lowest percentage of colocalization in the PFC (3.4% + 0.4) (Fig. 2d). Statisical analysis using two-way ANOVA confirmed a significant interaction between brain region and astrocyte colocalization (F(6,168) = 20.57, p < 0.0001). Confocal microscopy revealed that PPARγ weakly colocalizes in the processes of astrocytes but not in the cell body (Fig. 5d–f). Surprisingly, we found PPARγ does not colocalize with microglia in the adult mouse brain (Fig. 5g–i). Therefore, there was a significant difference between PPARγ colocalization with astrocytes and microglia (Tukey HSD, p < 0.0001).

### PPAR isotypes demonstrate similar cell type specificity in human brain

**Figure 6:**
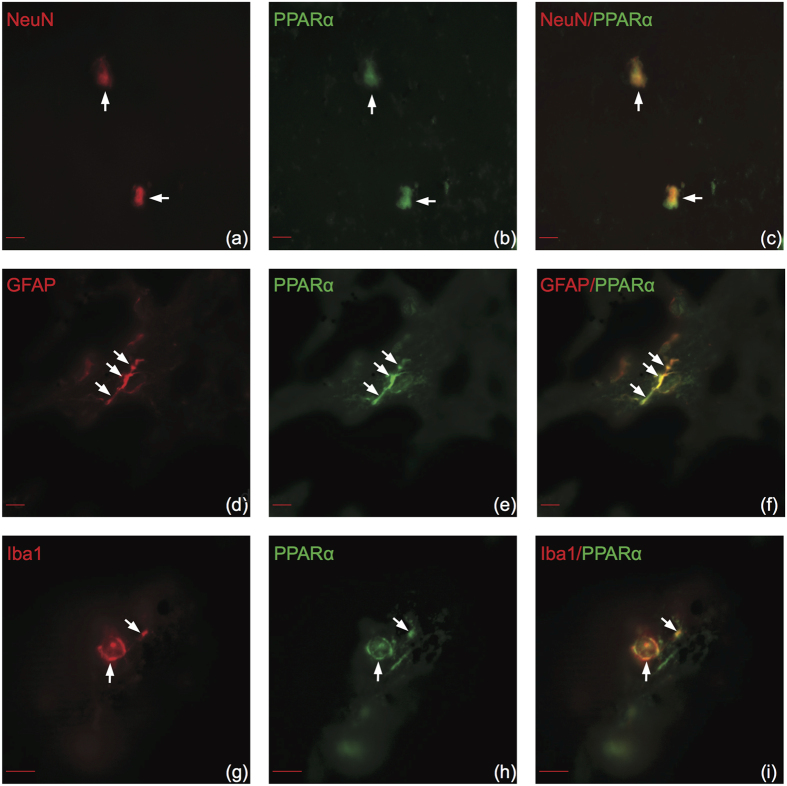
PPARα colocalizes with neurons, astrocytes and microglia in human brain. Representative images of cell type specific stains color-coded in red (left panels), PPARα color-coded in green (center panels), and merged images (right panels). (**a–c**) PPARα colocalizes with NeuN. (**d–f**) PPARα colocalizes with GFAP. (**g–i**) PPARα colocalizes with Iba1. Arrows indicate positive colocalization. Coronal sections correspond to the superior frontal gyrus (SU number 463, post mortem interval 51 h). Fluorescent microscope (63×), Scale bar (a-d) = 10 μm. Scale bar (g-i) = 20 μm.

**Figure 7:**
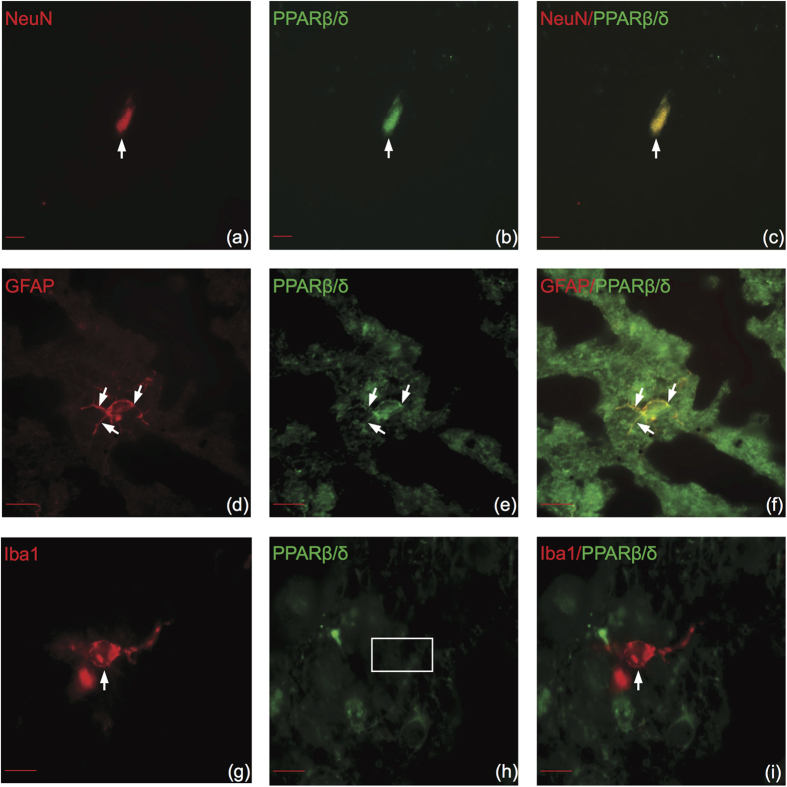
PPARβ/δ colocalizes with neurons and astrocytes but not microglia in human brain. Representative images of double immunofluorescence labeling of cell type specific stains color-coded in red (left panels), PPARβ/δ color-coded in green (center panels), and merged images (right panels). (**a–c**) PPARβ/δ colocalizes with NeuN. (**d–f**) PPARβ/δ colocalizes with GFAP. (**g–i**) PPARβ/δ does not colocalize with Iba1. Arrows indicate positive colocalization. Boxes indicate negative colocalization. Coronal sections correspond to the superior frontal gyrus (SU number 459, post mortem interval 9.5 h). Fluorescent microscope (63×), Scale bar (a-c) = 10 μm. Scale bar (d-i) = 20 μm.

**Figure 8:**
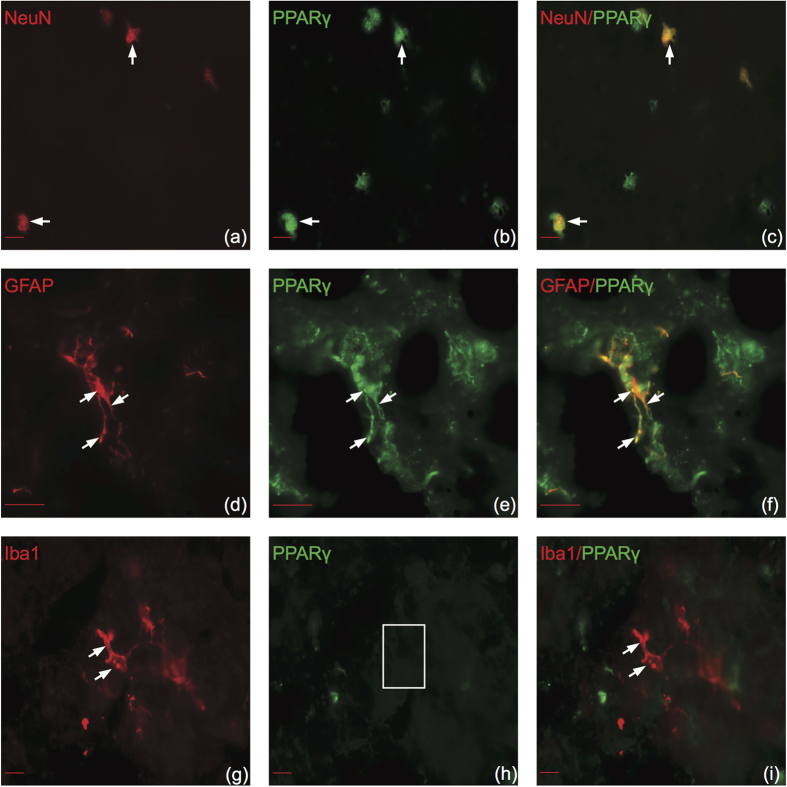
PPARγ colocalizes with neurons and astrocytes but not microglia in human brain. Representative images of double immunofluorescence labeling of cell type specific stains color-coded in red (left panels), PPARγ color-coded in green (center panels), and merged images (right panels). (**a–c**) PPARγ colocalizes with NeuN. (**d–f**) PPARγ colocalizes with GFAP. (**g–i**) PPARγ does not colocalize with Iba1. Arrows indicate positive colocalization. Boxes indicate negative colocalization. Coronal sections correspond to the superior frontal gyrus (SU number 442, post mortem interval 24 h). Fluorescent microscope (63×, (**a–d**,**g–i**)) and (100×, (**d–f**)). Scale bar (a-c,g-i) = 10 μm. Scale bar (d-f) = 20 μm.

There have been no previous studies investigating PPAR cell type specificity in adult human brain other than neuroblastoma cell lines^18^,^19^. Therefore, to determine if PPARs are expressed in similar cell types between mice and humans, we utilized double immunofluorescence in postmortem human superior frontal gyrus. PPARα colocalized with all cell types (Fig. 6a–i). PPARβ/δ colocalized with neurons (Fig. 7a–c) and astrocytes in white matter (Fig. 7d–f) but not microglia (Fig. 7g–i). PPARγ colocalized with neurons (Fig. 8a–c) and astrocytes (Fig. 8d–f) but not microglia in human brain (Fig. 8g–i). Overall, all PPAR isotypes displayed similar cell type specificity between adult mouse and human brain tissue.

### Specific PPAR isotypes colocalize with microglia after LPS treatment

**Figure 9:**
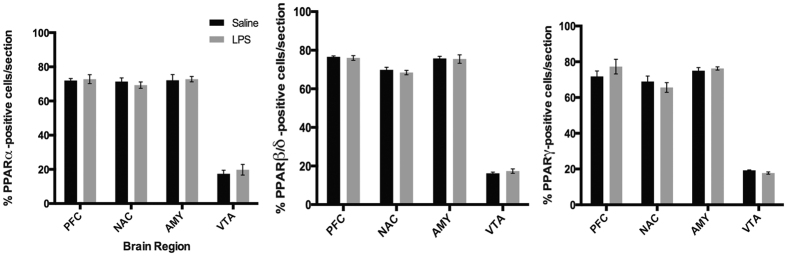
LPS administration does not change PPAR isotype expression in brain. Quantification of PPAR isotype distribution in subregions of the adult mouse brain. Data are represented as mean + SEM, n = 5 per group.

**Figure 10:**
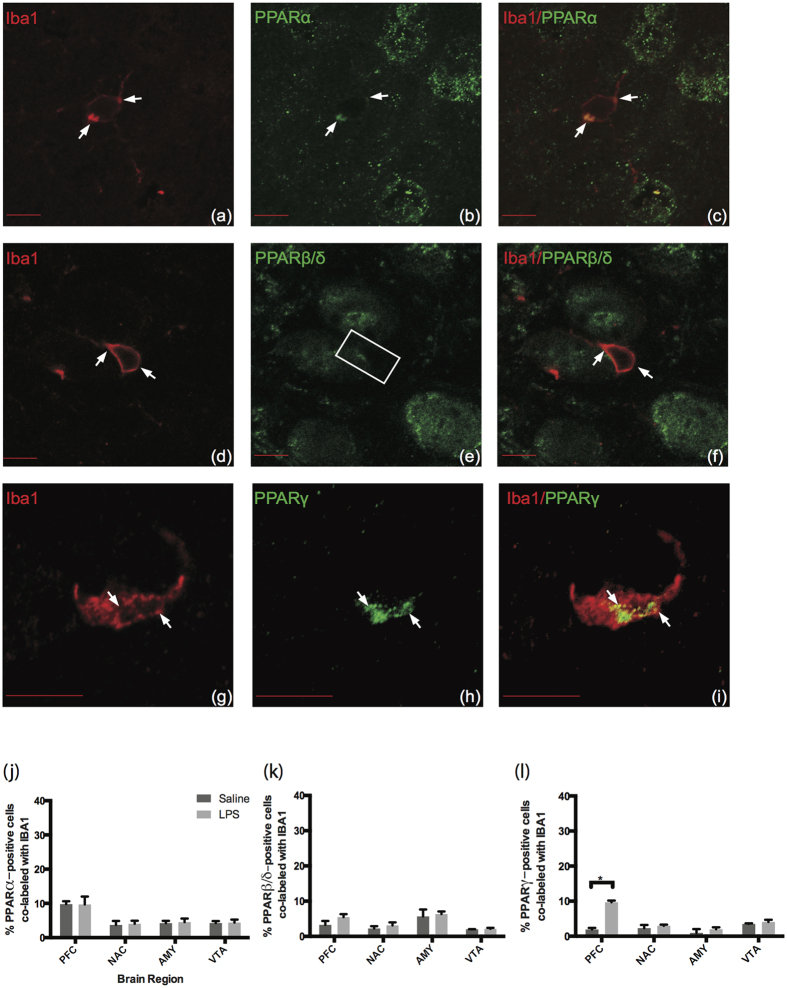
PPARα and PPARγ colocalize with Iba1 in mouse prefrontal cortex after LPS injection, but not PPARβ/δ. Representative images of Iba1 color-coded in red (left panels), PPAR isotypes color-coded in green (center panels), and merged images (right panels), confocal 63 × (a-f) and confocal 100 × (g-i), Scale bar = 10 μm. Arrows indicate positive colocalization and yellow in the merged images. Boxes indicate negative colocalization. (**a–c**) PPARα colocalizes with Iba1 after LPS. (**d–f**) PPARβ/δ does not colocalize with Iba1. (**g–i**) PPARγ colocalizes with Iba1 after LPS. (**j–l**) Quantification of microglia colocalization after LPS. Data are expressed as mean + SEM, n = 5 per group, *p < 0.001.

The observation that PPARγ did not highly colocalize with microglia in mouse or human brain was surprising since many studies observe a reduction in microglial activity after PPARγ agonist administration (for review see^4^). Moreover, in rat primary microglia cultures the constitutive expression of PPARγ is up-regulated by specific agonists and down-regulated during the process of activation induced by LPS, suggesting that the expression of this receptor is regulated and dependent on microglial functional state^22^. Therefore, we hypothesized that the cell type specificity of PPAR isotypes, in particular PPARγ, may also be tightly regulated and dependent on microglial functional state. To address this we first determined if LPS administration altered PPAR isotype protein expression. There was no observed change in PPAR isotype protein expression in any brain region after LPS (Fig. 9, two-way ANOVA, F(1,16) = 0.1854, p = 0.6725). Next, we investigated if LPS alters the cell-type specificity of PPAR isotypes, which would suggest dependence on microglial functional state. Confocal microscopy confirmed that PPARα and PPARγ colocalize with microglia after LPS treatment (Fig. 10a–c,g–i). PPARβ/δ still did not colocalize with microglia after LPS treatment (Fig. 10d–f). Quantification of PPAR isotype colocalization with microglia (Fig. 10j–l) revealed that only PPARγ had a significant increase in the number of colocalized microglia compared to saline controls in the PFC (t = 20.812, p < 0.0001). Additionally, we noted that PPARα was the only isotype to colocalize with microglia under both normal and LPS conditions, even though there was not a significant increase in PPARα-microglia colocalization after LPS.

## Discussion

PPAR agonists are promising treatments for various brain pathologies due to their anti-inflammatory, neuroprotective and anti-addictive properties^4^,^24^,^25^,^26^,^27^,^28^,^29^,^30^,^31^,^32^,^33^,^34^. Although PPARs in CNS cells have been extensively studied in rats and mice *in vitro*^9^,^14^,^15^,^18^,^19^,^22^, studies of cell type specific PPARs in brain tissue are still limited^5^,^9^,^20^. Moreover, to the best of our knowledge, there are currently no available studies investigating the cell type specificity of all PPAR isotypes in human brain tissue. Given that PPAR agonists are being tested in human clinical trials, it is surprising that there are no cell type specificity profiles defined for PPAR isotypes in human brain tissue. Specifically, the PPARγ agonist pioglitazone has been used in two small human trials, which found that pioglitazone administration resulted in cognitive and functional improvements in Alzheimer’s patients^33^,^34^. Despite the small sample sizes, these studies suggest that PPAR agonist administration in humans may offer a novel strategy for treating neurodegenerative diseases. Thus, our cell type specificity profiles in both animal models and human brain tissue will permit future studies not only to select PPAR agonists based on cell type specificity but also to determine how PPAR agonists provide neuroprotective effects in the mouse and human brain.

The present report fills this gap in knowledge by determining PPAR isotype cell type specificity in both the adult mouse and adult human brain as well as characterizing how PPAR isotype cell type specificity changes after LPS administration. Additionally, we provide a detailed, brain region-specific distribution map of PPAR isotype mRNA and protein to complement the cell type specificity profiles in brain regions important to addiction and neurodegenerative diseases. We found that (i) PPAR isotype mRNA and protein is ubiquitously expressed across brain regions, with higher expression in the PFC, NAC, and AMY compared to the VTA; (ii) PPARβ/δ and PPARα mRNA and protein are more highly expressed than PPARγ; (iii) PPARα is the only isotype to colocalize with all cell types; (iv) LPS administration does not alter PPAR isotype protein expression in brain tissue; (v) LPS administration does alter cell type specificity of PPARγ. The ubiquitous distribution and unique cell type specificities of PPARs in the CNS may provide additional insight into how PPAR agonists result in neuroprotective and anti-inflammatory effects.

Previous studies have investigated PPAR isotype mRNA and protein distribution in the rat CNS^5^,^9^,^10^,^20^. On the mRNA level, coarse brain regions as well as more specific brain regions—such as the caudate putamen, hippocampus, hypothalamus, and thalamus—have been profiled^8^,^10^,^11^. However, PPAR isotype distribution in key regions for neurodegenerative disorders and addiction (i.e. prefrontal cortex, amgydala and ventral tegmental area) has not been profiled. Similarly, PPAR isotype protein expression has been investigated but the expression of PPAR isotypes in the amygdala and prefrontal cortex remains unknown^5^,^20^. Thus, our data in collaboration with previously published literature provides a more detailed and complete map of PPAR isotype distribution on both the mRNA and protein level.

The general order of abundance across all brain regions for PPAR mRNA and protein was PPARβ/δ > PPARα ≥ PPARγ, consistent with what has been observed in coarse brain reigons of the rat CNS^5^. We observed novel protein expression of all isotypes in the prefrontal cortex and amygdala as well as presence of immunoreactivity in the ventral tegmental area for PPARα and PPARγ^5^,^20^. Our findings that all PPARs are expressed in the prefrontal cortex, nucleus accumbens, amygdala and ventral tegmental area are consistent with a possible role for PPARs in the reward circuits involved in addiction, which involve the ventral striatal circuitry and extended amgydala circuitry^35^,^36^. Taken together, the results (Table 1) provide additional evidence that all PPAR isotypes are present but variably expressed throughout the adult mouse CNS.

Although PPAR agonist administration (α, γ, dual α/γ, and pan-α/β/δ/γ) results in cell type specific changes in the CNS, there are no comprehensive studies of PPAR isotype cell type specificities in the adult rat or adult mouse CNS^21^,^22^,^37^,^38^. Moreover, the few available studies in rat find conflicting results for PPAR isotype protein cell type specificity, most likely due to differences in animal age, model system, and techniques utilized (Supplemental Table S1). We show that in both the adult mouse CNS and adult human brain, PPARα colocalizes with all cell types, PPARβ/δ only colocalizes with neurons in grey matter, and PPARγ colocalizes with neurons and astrocytes but not microglia without administration of LPS. Given that PPAR agonist administration results in robust inhibition of inflammatory gene expression, we expected a majority of PPAR isotype positive cells to colocalize with microglia. However, our immunohistochemical analysis only demonstrated weak colocalization between PPARα and microglia under normal conditions. Furthermore, the percentage of microglia that colocalized with PPARα or PPARγ after LPS treatment was less than 10% in all adult mouse CNS brain regions. This suggests that microglial colocalization with PPARs is weak, even after a strong neuroimmune response. However, this weak effect may have been due to the short time course for LPS treatment.

Notably, we saw a strong neuronal signature of PPAR isotypes with over 90% of neurons colocalizing with each PPAR isotype across brain regions in the adult mouse CNS. A previous study from our group also observed a strong neuronal signature for gene expression changes of all PPAR isotypes in the prefrontal cortex and amygdala after PPAR agonist administration in mice^21^. Ferguson and colleagues observed that only PPARα and dual-PPARα/γ agonists resulted in enrichment of genes that are preferentially expressed in astrocytes, corroborating this study’s findings that only PPARα and PPARγ colocalize with astrocytes in grey matter. Moreover, they found a lack of genes associated with microglia after PPAR agonist administration, which is consistent with the weak immunoreactivity we observed between PPARα and microglia under normal conditions. Overall, the corroboration between the gene expression data and our cell type specificity profiles suggests that PPAR agonists may not be providing neuroprotective effects by regulating microglial responses. Instead PPAR agonists may be targeting other cell types, to induce to neuroprotective changes and protect neurons against oxidative stress-induced cell death under various conditions such as ischemia^39^, traumatic brain injuries^40^ or neurodegenerative disease^24^ in both the adult mouse and human CNS.

Still, a remaining question is how PPARγ agonists modulate microglial activity in the CNS because our data indicate that PPARγ does not colocalize with microglia under normal conditions and only weakly colocalizes after a strong neuroimmune response. Moreover, PPARγ did not colocalize with microglia in human brain, most likely because the tissue utilized in this study did not have an inflammatory response. An important question is if cell type specific changes induced by PPARγ agonists are due to PPARγ-dependent or -independent mechanisms. A recent study found that 15d-PGJ2-induced astrocyte-mediated neuroprotection may not be a PPARγ-mediated pathway because knockdown of PPARγ, a well-characterized 15d-PGJ2 target, did not alter 15d-PGJ2 non-cell autonomous neuroprotection in astrocytic culture^41^. Additionally, several PPARγ agonists of the thiazolidinedione (TZD) family failed to induce neuroprotection suggesting that the neuroprotective and anti-inflammatory effects of PPAR agonist administration may be cell type specific or even independent of PPARγ^41^. This is consistent with the finding that after a repeated brain injury pioglitazone reduced damage and inflammation but PPARγ and PPARγ target gene expression was not induced^42^. Additional experiments utilizing neuroimmune stimulators and a range of PPARγ antagonists in cell type specific cultures and *in vivo* are needed to elucidate how changes in glial activation occur after PPAR agonist administration.

In summary, we define the distributions of PPAR isotype mRNA and protein in specific brain regions important for neurodegenerative diseases and addiction. We found that all PPARs are expressed in multiple brain regions. Each PPAR isotype has a distinct cell type specificity profile, with all PPAR isotypes highly expressed in neurons. The strong neuronal signature of PPAR isotypes in the adult mouse and human brain was unexpected and may be important for determining how PPAR agonists provide beneficial neuroprotective and anti-inflammatory effects. In concert with previously published literature, this characterization will aid researchers studying CNS disorders that are responsive to PPAR agonists by providing a distribution and cell type specificity profile across mouse and human brain tissue. This will enable future studies to selectively choose PPAR agonists based on brain region expression and cell type specificity to provide more targeted neuroprotective treatments. Moreover, it will provide the necessary foundation for understanding how PPAR agonists alter specific cell types and cell signaling in the human brain to provide novel therapeutic effects in the treatment of neurodegenerative diseases and addiction.

## Methods

### Animals

Male C57BL6/J mice (8 weeks of age, original breeders were purchased from Jackson Laboratories, Bar Harbor, ME) were used for all experiments. All experiments were approved by The University of Texas at Austin Institute for Animal Care and Use Committee and conducted in accordance with NIH guidelines with regard to use of animals in research.

### Brain Collection

Deeply anesthetized mice for IHC (n = 5) were transcardially perfused at room temperature (RT) with 0.9% saline followed by freshly prepared 4% paraformaldehyde^43^ in phosphate-buffered saline (PBS) and then post fixed in 4% PFA at 4 °C for 24 h followed by cryoprotection for 24 h at 4 °C in 20% sucrose. Brains utilized for qPCR (n = 7) experiments were freshly harvested then snap frozen in liquid nitrogen. Frozen brains for both IHC and qPCR were molded in a plastic mold containing optimum cutting temperature compound (OCT, VWR, Radnor, PA) and quickly frozen in isopentane on dry ice.

### qPCR

Micropunches were taken as previously described^44^. The following coordinates, anterior-posterior distance from Bregma, were utilized: PFC (+3.2 mm to +1.8 mm); NAC (+1.8 mm to +0.6 mm); AMY (−0.9 mm to −1.8 mm); VTA (−2.8 mm to −3.4 mm). Total RNA was isolated from brain region micropunches using the MagMAX-96 Total RNA Isolation Kit (Life Technologies, Grand Island, NY). Total RNA was quantified on a NanoDrop 8000 spectrophotometer (Thermo Fisher Scientific, Grand Island, NY) and assessed for quality using the Agilent TapeStation (Agilent Technologies, Santa Clara, CA). All samples passed quality control measures (RIN > 8). Reverse transcription was performed using the Applied Biosystems High Capacity cDNA reverse transcription kit (Applied Biosystems, Grand Island, NY). PCR amplification was performed using TaqMan Universal PCR Master Mix and primer pairs and probes (Thermo Fisher Scientific, Grand Island, NY). The sequences of the TaqMan PCR assays used are shown in Supplemental Table S3 as well as sequence alignment and differences for the probes used. Relative quantification of mRNA levels was determined using qbase software as previously described^43^,^45^. The following genes were checked as endogenous controls: Ppia, GAPDH, β-Actin, Gusb, 18S. β-Actin was selected as the endogenous control to normalize target gene mRNA levels due to its lack of variability between brain regions and samples.

### Antibodies

Primary antibodies: rabbit anti-PPARα, Abcam (Cambridge, MA), 1:50; rabbit anti-PPARβ/δ, Pierce (Rockford, IL), 1:100; rabbit anti-PPARγ, Abcam, 1:20; mouse anti- neuronal nuclei-neuron specific nuclear protein (NeuN), Millipore (Billerica, MA), 1:500; mouse anti-glial fibrillary protein (GFAP), NeuroMab, 1:300; goat anti-ionized calcium-binding adapter 1 (Iba1), Abcam, 1:300. Secondary antibodies: goat anti-rabbit 488, donkey anti-rabbit 488, goat anti-mouse 594 or donkey anti-goat 568, Invitrogen (Grand Island, NY), 1:1,000. For complete list of antibodies see Supplemental Table S2.

### Antibody Specificity

We used three sets of controls to determine specificity of our antibodies used in subsequent stains. (1) Primary antibody controls, when tissue is available: shows the specificity of primary antibody binding to the antigen. (2) Secondary antibody controls: shows the label is specific to the primary antibody. (3) Label controls: shows the labeling is the result of label added during the procedure and not endogenous labeling or reaction products.

#### Primary antibody controls

With available PPARα knockout tissue, we tested the specificity of the PPARα antibody. As shown in Supplemental Fig. S1A, there was no immunoreactivity of the PPARα antibody in a PPARα knockout—confirming specificity of this antibody. As for PPARγ, Sarruf *et al*. tested five commercially available PPARγ antibodies (including the Abcam rabbit anti-PPARγ used in this study) and found similar immunoreactivity patterns and a reduction in signal in floxed PPARγ knockouts using the antibody used in this study^46^,^47^. If knockout tissue is not easily obtainable or previously published, one can also use immunoblots to determine whether the primary antibody can bind to a single protein of the correct molecular weight. For PPARβ/δ other researchers have confirmed the specificity of the primary antibody used in this study using western blots^15^,^48^.

#### Secondary antibody controls

We tested specificity of the secondary antibody by performing either replacement of the primary antibody with only the serum of the appropriate species or elimination of primary antibody. As shown in Supplemental Fig. S1C, no immunostaining was detected under either of these conditions for each secondary antibody used in the study.

#### Labeling antibody controls

Labeling controls are necessary to prevent autofluorescence from being mistaken for primary antibody specific signal. We performed a labeling control, which include a sample of tissue section that is incubated in all of the buffers and detergents used in the experiment but no antibodies, enzymes, or dyes. We then evaluated the labeling in control slides, using a confocal microscope with the intensity setting and exposure times that are the same as those used for examining a primary antibody containing and labeled sample. As shown in Supplemental Fig. S1B, for each detergent condition we observe no autofluorescence in mouse tissue. Due to high levels of autofluorescence in human tissue, we used an autofluorescence eliminator product (Millipore #2160, Millipore) to avoid autofluorescence signal problems. As shown in Supplemental Fig. S1B, No immunostaining was detected for the labeling control.

In addition to the control experiments run that show specificity of the antibodies, the antibodies for PPARα, PPARβ/δ, and PPARγ in the present study have been widely used and their specificities have been previously validated by immunoblot, immunocytochemistry and immunohistochemistry by independent researchers in mice and humans^15^,^47^,^49^,^50^,^51^,^52^.

### Double Immunofluorescence

The following coordinates were chosen for all immunohistochemistry experiments: PFC, (Bregma +2.8 to +2.24), NAC (Bregma +1.10 to +0.8), AMY (Bregma −1.20 to −1.60), and VTA (Bregma −3.08 to −3.4). Dual immunofluorescence was performed simultaneously on each section per brain region: PPAR isotype (α, β/δ, γ) and NeuN for neurons, GFAP for astrocytes, Iba1 for microglia. Briefly, 30 μm sections were permeabilized in optimized detergent (0.5% Triton-X-100 or 0.1% Tween-20), then blocked in 10% goat or donkey serum (Equitech-Bio, Kerrville, TX) for 1 h at RT. Sections were then incubated with primary antibodies overnight at 4 °C. Following three washes in PBS, sections were then incubated with the corresponding secondary antibody for 2 h at RT. Finally, sections were mounted in 0.2% gelatin, dehydrated, and cover slipped with a DAPI (4′,6-diamidino-2-phenylindole)-containing mounting medium (Vector Labs, Burlingame, CA).

### Human Tissue Double Immunofluorescence

Human autopsy brain samples were obtained from the New South Wales Tissue Resource Centre at the University of Sydney. Tissue was collected as previously described^53^. Briefly, cases were matched as closely as possible by age, gender, post-mortem interval (PMI) and brain pH (see Supplemental Table S4). Diagnoses were confirmed by physician interviews, review of hospital medical records, questionnaires to next-of-kin, and from pathology, radiology and neuropsychology reports. Cases were also chosen on the basis that agonal hypoxia did not appear to have differed significantly from the study group. Moreover, none of the brains showed evidence of hypoxic encephalopathy, further suggesting that agonal hypoxia was minimal. We did not accept cases that suffered prolonged agonal states.

Fresh frozen samples of the superior frontal gyrus were collected from each postmortem sample (Controls, Male, Caucasian, Age 48–71, n = 4, Case information in Supplemental Table S4). Brain tissue was sectioned at 9 μm intervals in the coronal plane and mounted on slides. We adapted a previously described protocol^54^ for non-fixed postmortem adult human brain tissue. Briefly, 9 μm sections were fixed using 4% PFA on ice. Sections were then permeabilized in PBS containing 0.5% Triton-X and blocked in 10% goat or donkey serum (Equitech-Bio Inc., Kerrville, TX) for 1 h at RT. Sections were then incubated with primary antibodies overnight at 4 °C. Following three washes in PBS, sections were incubated with the corresponding secondary antibody for 1 h at RT. Tissue was then washed in 70% ethanol followed by a 10 minute incubation with an autofluorescence eliminator reagent (Millipore #2160, Millipore). Sections were rinsed in 70% ethanol, dehydrated, and cover slipped with a DAPI-containing mounting medium (Vector Labs. Burlingame, CA). Staining for microglia required a longer fixation (48 h at 4 °C) and a longer primary incubation (72 h at 4 °C).

### Microscopy

Quantification of PPAR isotype positive cells was performed using a Zeiss Axiovert 200 M fluorescent light microscope (Zeiss, Thornwood, NY) equipped with an Axiocam b/w camera. Brain regions were identified using a mouse brain atlas as previously described^55^. Bilateral images of the PFC (Bregma +2.8 to +2.24), NAC (Bregma +1.10 to +0.8), AMY (Bregma −1.20 to −1.60), and VTA (Bregma −3.08 to −3.4) were captured using a 20x objective. We quantified PPAR isotype distribution, defined as the number of PPAR isotype positive cells per section divided by total cell count (i.e. DAPI). Immunoreactivity was defined as follows: high (>66%), moderate (33–66%), or weak (<33%). Briefly, images were quantified using ImageJ 1.42q^56^ by an experimenter blind to condition. Brain regions were traced, separated into quadrants and overlaid with a 10-mm^2^ grid. Within each quadrant, four representative grids were chosen randomly for counting then summed to give % PPAR positive cells per section. Cell counts were performed within each grid by ImageJ plug-in ITCN (http://rsb.info.nih.gov/ij/plugins/itcn.html). For all cell quantifications, cells were counted in both hemispheres for a given region and summed. Total cell counts for each animal were then averaged and presented as % PPAR positive cells per section. As a control to ensure that the PPAR expression quantification performed on a fluorescent light microscope (Fig. 2a) was accurate, we quantified one isoform’s (PPARβ/δ, n = 4) expression on a Zeiss confocal microscope. As shown in Supplemental Fig. S2, the percentage of PPARβ/δ per section quantified on a confocal microscope rather than a fluorescent light microscope yielded similar results, (t = 0.2846, p = 0.9932).

Quantification of different PPAR isotypes in neurons (NeuN), astrocytes (GFAP) and microglia (Iba1) were performed using a Zeiss 710 laser-scanning confocal microscope. Brain regions were identified using the mouse brain atlas^57^. Images were quantified bilaterally within fixed area frames; PFC (box, 645 μm × 645 μm), NAC (circle, 575 μm diameter), and AMY (circle, 675 μm diameter), and VTA (box, 275 × 275 μm). Confocal images were captured bilaterally at 20× in two sections. PPAR isotype and cell type (NeuN/GFAP/Iba1) dual-labeled cells were quantified in the PFC (Bregma +2.8 to +2.24), NAC (Bregma +1.10 to +0.8), AMY (Bregma −1.20 to −1.60), and VTA (Bregma −3.08 to −3.4) using ImageJ plug in ITCN for identification of immunopositive cells (green somatic labeling for PPAR isotypes). Cells were considered co-localized when they expressed both green (PPAR isotype) and red (NeuN/GFAP/Iba1) fluorescence within the same z-stack. For PPAR isotypes/NeuN co-expressing cells PPAR was found within the extra-nuclear area, while NeuN was localized to the nucleus, resulting in a green cell body that surrounds a red nucleus. For PPAR isotypes/GFAP positively co-expressing cells, PPAR isotype immunoreactivity was found in the astrocytic processes, while GFAP was identified with astrocyte-positive morphology wrapping around a nucleus visualized by DAPI. Thus, in the same z-stack PPAR isotypes/GFAP positively co-expressing cells were visualized as yellow/orange processes resulting from an overlap of the red and green secondary labeling. For PPAR isotypes/Iba1 positively co-expressing cells, PPAR isotype immunoreactivity was found in the microglial processes, while Iba1 was identified with microglia-positive morphology wrapping around a nucleus visualized by DAPI. Therefore, in the same z-stack PPAR isotype/Iba1 positively co-expressing cells were visualized as yellow/orange processes and a red cell body (as seen in Fig. 2g–i). Within brain regions, PPAR isotypes and cell type positive cells were counted by adapting a previously reported double-blinded non-stereological method, using ITCN for cell counts^55^. Within brain regions, PPAR isotype and cell types (NeuN/GFAP/Iba1) were counted as were cells co-expressing PPAR isotypes/NeuN, PPAR isotypes/GFAP, or PPAR isotypes/Iba1. These counts were then used to determine both the total number and percentage of PPAR isotype positive cells that expressed NeuN, GFAP, or Iba1. For all cell quantifications, cells were counted in both hemispheres of a given region in each section and summed. Cell counts for two sections were averaged and are presented as percentage of co-expressing cells per section. Representative colocalization images were captured at 63× magnification on a Zeiss 710 laser-scanning confocal microscope (Zeiss, Thornwood, NY) unless otherwise listed. A total of 12 representative colocalization images were taken for each isotype and cell type marker. Only representative images were altered for contrast/brightness. No images used for quantification were altered in any way as to not bias colocalization quantification.

### LPS treatment

LPS (strain O111:B4, Sigma, St. Louis, MO) dissolved in saline was intraperitoneally injected at a dose of 5 mg/kg i.p. in volume 0.1 ml/10 g of body weight of male C57BL/6 J mice (n = 5). The dosage of LPS was based on a previous study that demonstrated that this single high dose of LPS actively induces persistent inflammation and progressive neurotoxicity over 10 months in the adult mouse^29^. Brains were collected at 6 hours post LPS injection and prepared for IHC as above. The 6-hour time point was selected because brain cytokines have been shown to remain at stable and elevated levels at this time point after a single high dose of LPS^29^.

### Statistical Analyses

Five mice (n = 5) were utilized for double immunofluorescence under normal conditions and LPS treatment. Human double immunofluorescence included four samples for each isotype (n = 4). Seven mice (n = 7) were utilized for qPCR, data are expressed as mean fold change +/− SEM.

All statistical analyses were carried out using GraphPad (San Diego, CA). Data distribution was verified using R software built in stats packages (Vienna, Austria). To determine if data was normally distributed the following tests were done: (1) plot histogram of data; (2) Q-Q plot. qPCR data was analyzed using BIORAD gene expression software (Hercules, CA). qPCR statistical significance was analyzed using a one-way ANOVA, across all brain regions with Tukey HSD post hoc analysis (α = 0.95). For PPAR distribution, data is presented as the average percentage of cells expressing each PPAR isotype per section (+/− SEM). PPAR distribution was assessed using a two-way ANOVA with Tukey HSD post hoc analysis (α = 0.95). For dual-labeled immunofluorescence quantification, data is presented as the mean percentage of PPAR isotype cells co-expressing cell type markers (NeuN,GFAP,Iba1) (+/− SEM). To analyze the percentage of co-labeled PPAR isotypes/cell type specific positive cells, a two-way ANOVA was utilized with Tukey HSD post hoc analysis (α = 0.95). For PPAR distribution after LPS administration, data is presented as the average percentage of cells expressing each PPAR isotype per section (+/− SEM). A Student’s t-test (α = 0.95, two-tail) was used to compare PPAR expression by fluorescent microscope versus confocal microscope for each brain region individually. A Student’s t-test (α = 0.95, two-tail) was used to compare PPAR isotype/microglia colocalization in LPS versus saline groups for each brain region individually.

## Additional Information

**How to cite this article**: Warden, A. *et al*. Localization of PPAR isotypes in the adult mouse and human brain. *Sci. Rep.* **6**, 27618; doi: 10.1038/srep27618 (2016).

## Supplementary Material

Supplementary Information

## Figures and Tables

**Table 1:** Summary IHC Expression.

	PPARα	PPARβ/δ	PPARγ
Prefrontal Cortex	+++	+++	++
Nucleus Accumbens	++	++	++
Amygdala	++	++	++
Ventral Tegmental Area	+	+	+

Distribution of PPAR isotype protein expression in subregions of the adult mouse brain. Immunoreactivity was defined as the proportion of labeled PPAR isotype cells in a given brain structure: high (+ + +), > 67% of cells positive for PPAR isotype; moderate (+ +), between 34% and 66% of cells positive for PPAR isotype; weak (+), < 33% of cells positive for PPAR isotype.
