# Supplementary material for: Localization of PPAR isotypes in the adult mouse and human brain
Source: Sci Rep. 2016 Jun 10;6:27618. doi: 10.1038/srep27618 (PMC4901333; doi:10.1038/srep27618)
Supplement: Supplementary Information [file srep27618-s1.pdf]

Localization of PPAR isotypes in the adult mouse and human brain

Warden, A.*, Truitt, J., Merriman, M., Ponomareva, O., Jameson, K., Ferguson, L.B., Mayfield, R.D., Harris, R.A

Supplementary Material.

A)

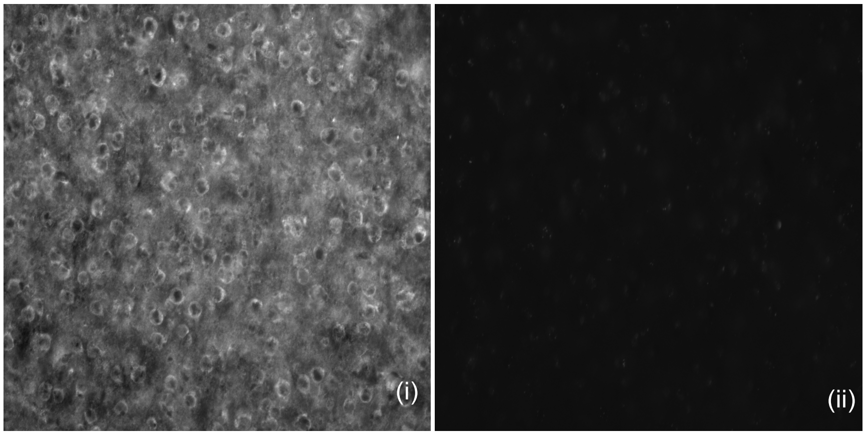

B)

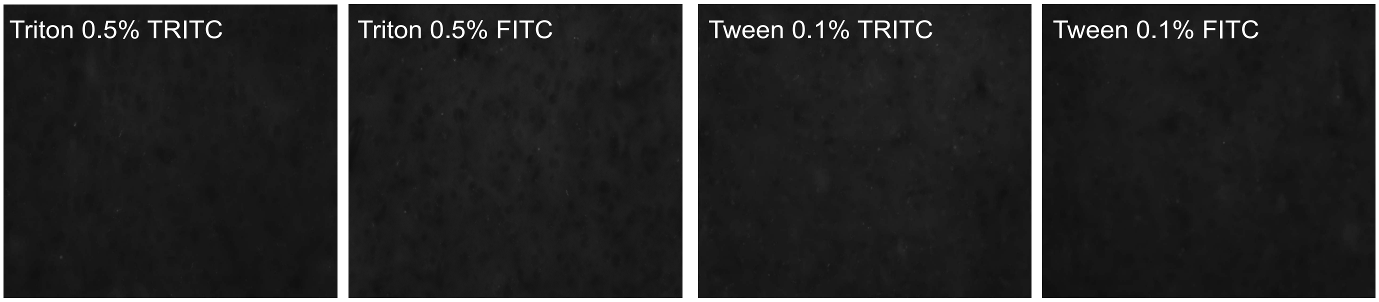

C)

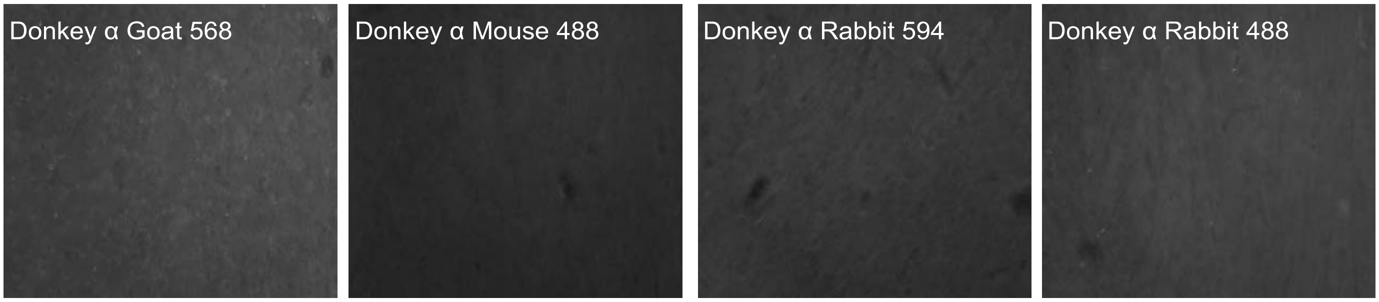

Supplementary Fig S1: Quality Controls for Immunohistochemical Quantification. A) PPARα knockout tissue staining control. (i) PPARα stained in the prefrontal cortex of a C57Bl6/J male mouse. (ii) PPARα stained in the prefrontal cortex of a male PPARα knockout mouse. Image taken on fluorescent microscope (20x). B) Labeling staining controls for all staining conditionings in nucleus accumbens of a C57Bl6/J male mouse--primary antibody and secondary antibody omitted, 10% donkey serum only. Image taken on fluorescent microscope (20x).
C) Secondary controls for all secondaries in prefrontal cortex of a C57Bl6/J male mouse--primary antibody omitted, 10% donkey serum and appropriate secondary. Image taken on a fluorescent microscope (20x).

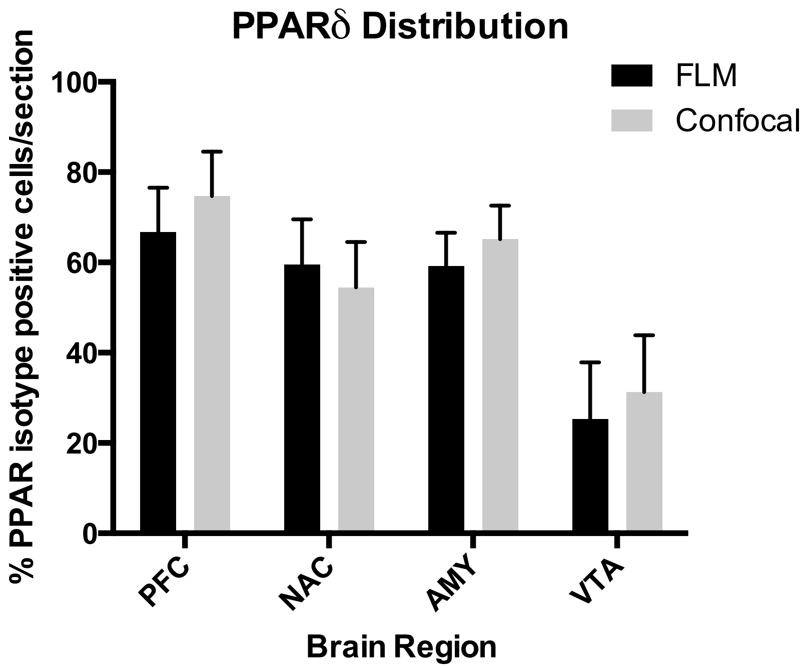

Supplemental Fig. S2: Comparison of PPARβ/δ expression by confocal microscope or fluorescent light microscope. No significant difference was found between quantification methods. All images taken (20x). Data are represented as mean (+/- SEM).

Isotype	Cell type	Kainu et al., 1994 ¥	Braissant et al., 1996 ¥	Cullingford et al., 1998*	Bernardo et al., 2000 *	Cristiano et al., 2001*	Moreno et al., 2004¥	Woods et al., 2003*¥	Cimini et al., 2005*	Inestrosa et al., 2005*	Sarruf et al., 2009¥	Present Results
	Neurons	+		+					+			+
	Astrocytes	-	-	+		+	+					+
PPARα	Microglia											+
	Neurons		+	+				+	+			+
	Astrocytes			+		+	-	-				-
PPARβ/δ	Microglia			+								-
	Neurons			+					+	+	+	+
	Astrocytes		-	+		+	-					+
PPARγ	Microglia				+							-

*=in vitro

¥=brain tissue

Supplemental Table S1: Summary of previous PPAR literature investigating cell type specificity. Symbols: +, PPAR isotype positively colocalizes with cell type; —, PPAR isotype does not colocalize with cell type; +/-, evidence of both positive and negative colocalization, blank, study did not investigate. Our study found that PPARα was in all cell types. PPARβ/δ was found only in neurons and astrocytes in white matter but not astrocytes in grey matter or microglia. PPARγ colocalized with neurons and astrocytes but not microglia.

Antibody	Host	Class	Company	Catalog #	Concentration
Primary:					
GFAP	Mouse	monoclonal	NeuroMab	Clone N206A/8	1:300
Iba1	Goat	polyclonal	Abcam	ab5076	1:300
NeuN	Mouse	monoclonal	Millipore	MAB377	1:500
PPARα	Rabbit	polyclonal	Abcam	ab8934	1:50
PPARβ/δ	Rabbit	polyclonal	Pierce Antibodies	PA1823A	1:100
PPARγ	Rabbit	polyclonal	Abcam	ab19481	1:20
Secondary:					
Alexa Fluor 488	Goat-anti rabbit	polyclonal	Invitrogen	A11034	1:1000
Alexa Fluor 488	Donkey anti-rabbit	polyclonal	Invitrogen	R37118	1:1000
Alexa Fluor 594	Goat anti-mouse	polyclonal	Invitrogen	A11005	1:1000
Alexa Fluor 568	Donkey anti-goat	polyclonal	Invitrogen	A10042	1:1000

Supplemental Table S2: Antibody Information

A)

Isoform	Taqman Assay ID	Refseq ID	Exon Boundary	Assay location	Amplicon length
PPAR alpha	Mm0440937	NM_001113418.1 NM_011144.6	4-5	712	115
PPAR delta	Mm00803134	NM_011145.3	5-6	696	68
PPAR gamma	Mm01184322	NM_011146.3	5-6	669	101

B)

```
PPARalpha      AACTATTCGGCTGAAGCTGGTGTACGACAAGTGTGATCGGAGCTGCAAGATTCAGAAGAA
PPARgamma      ----------------------------AGGTGTGATCTTAACTGCCGGATCCACAAAAA
PPARdelta      --------------------------------------------------------AGAA
                                                                       * **

PPARalpha      GAACCGGAACAAATGCCAGTACTGCCGTTTTCACAAGTGCCTGTCTGTCGGGATGTCACA
PPARgamma      AAGTAGAAATAAATGTCAGTACTGTCGGTTTCAGAAGTGCCTTGCTGTGGGGATGTCTCA
PPARdelta      GAACCGCAACAAGTGTCAGTACTGCCGCTTCCAGAAGTGCCTGGCACTCGGCATGTCGCA
                *   * ** ** ** ******** ** ** ** ********  *  * ** ***** **

PPARalpha      CAATGCAATTCGCTTTGGAAGAATGCCAAGATCTGAAAAAGCAAAACTGAAAGCAGAAAT
PPARgamma      CAATGCCATCAGGTTTGGGCGGATGCCACAGGCCGAGAAGGAGAAGCTGTTGGCGGAGAT
PPARdelta      CAACGCTATCCGCTTTGGACGGATGCCGGAGGCCGAGAAGAGGAAGCTGGTGGCGGGGCT
               *** ** **  * *****  * *****     * ** **    ** ***   ** *   *

PPARalpha      TCTTACCTGTGAACACGACCTGAAAGATTCGGAAACTGCAGACCTCAAATCTCTGGG
PPARgamma      CTCCA---GTGATATCGACCAGCTGAACCCAGAGTCTGCTGATCTGCGAGCCCTG--
PPARdelta      GACTGCCAGCGAG--------------------------------------------
                       * **
```

Supplemental Table S3: A) Taqman assays information. B) Sequence alignment of the total amplicons of the three PPAR Taqman assays with the approximate probe location (highlighted in yellow), and 13 bases on either side of the assay location shown in blue—since the total length of Taqman probes are between 18-25 bases.

SU #	Age	Gender	Ethnic origin	Classification	Brain weight	DSMIV	Volume calc	Brain pH	PMI	Frozen tissue
463	73	Male	Caucasian	Control	1390	Control	1361	6.82	51	Left
451	73	Male	caucasian	Control	1380	Control	1380	6.8	48	Right
442	63	Male	caucasian	Control	1570	Control	1569	6.94	24	Right
459	64	Male	caucasian	Control	1390	Control	1331	6.94	9.5	Right

SU #	Clinical Cause of Death	Macroscopic diagnosis	Microscopic diagnosis	Liver Pathology	Toxicology	Smoking history	Smoking
463	Congestive cardiac failure, atrial fibrillation, ischemic heart disease	1. Small old infarcts in the medial occipital lobe and the posterior hippocampus.	1. Small old infarcts in the medial occipital lobe and the posterior hippocampus. 2. A few microinfarcts in the cerebellum .	N/A	N/A	25 per day X 40 years	Yes
451	Dilated cardiomyopathy, ischemic heart disease.	Normal brain	Normal brain	N/A	N/A	Smoked all his life - moderate	Yes
442	Atherosclerotic coronary heart disease	Normal brain	Normal brain	Macrovesicular steatosis	Atenolol: <1mg/L Irbesartan: 0.8mg/L Lignocaine: 1.7mg/L	10 cigarettes per day for majority of life.	Yes
459	Ischemic heart disease	Normal brain	Small old hemorrhagic infarct in cerebellum.	Congestion and steatosis	not provided with PM	Heavy smoker. Smoked all his life.	Yes

Supplemental Table S4: Human Tissue Cohort Information for Human Immunofluorescence. All case information has been previously published[53].
